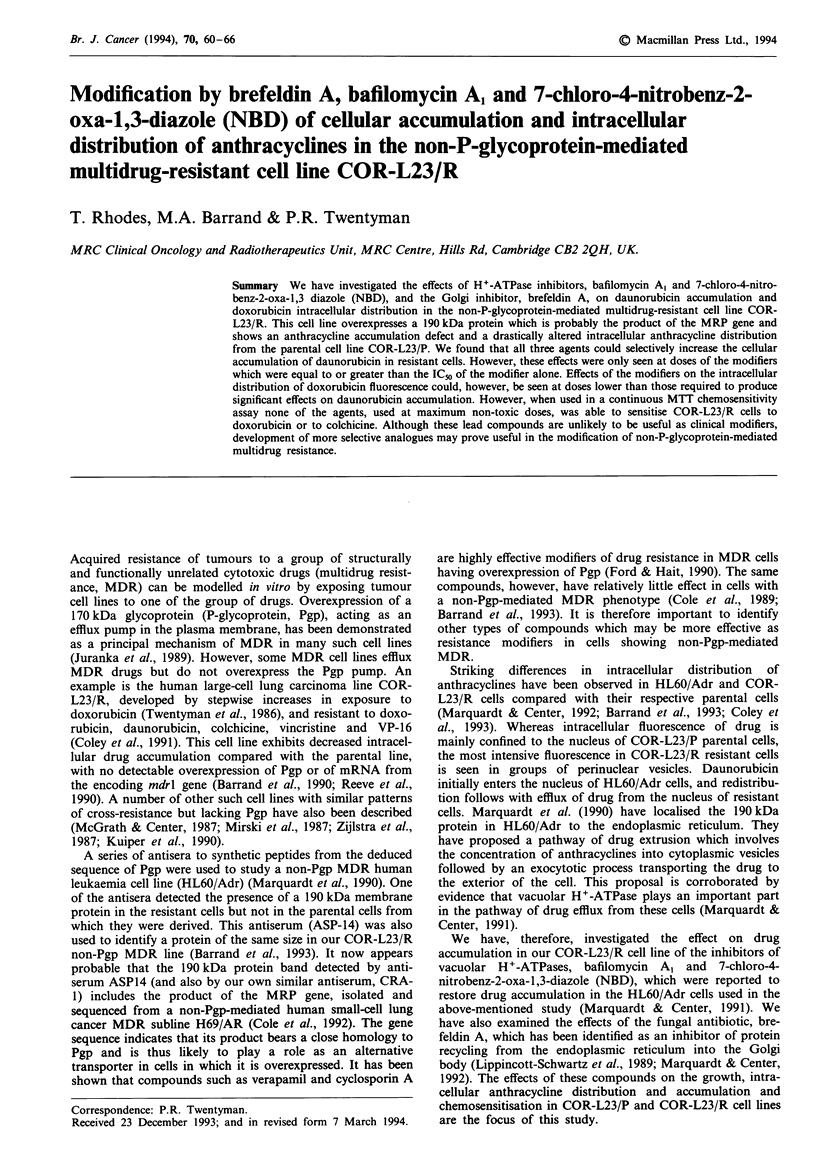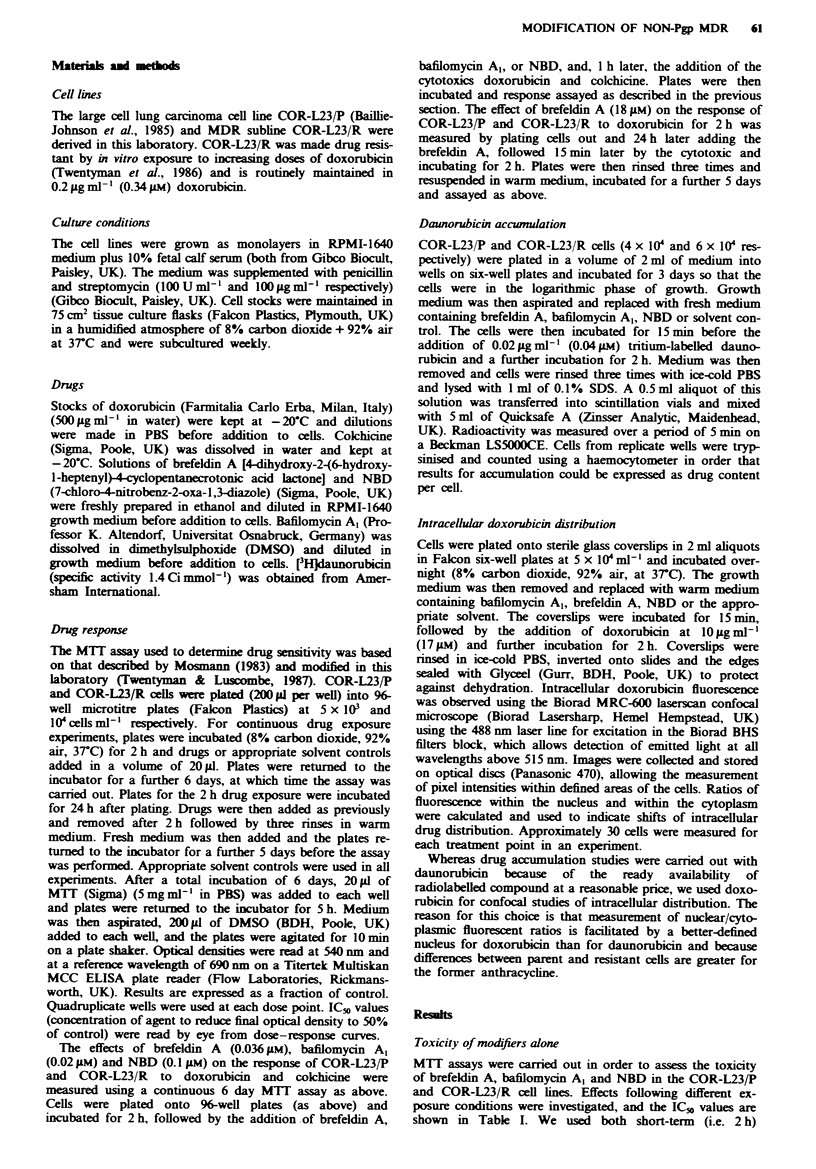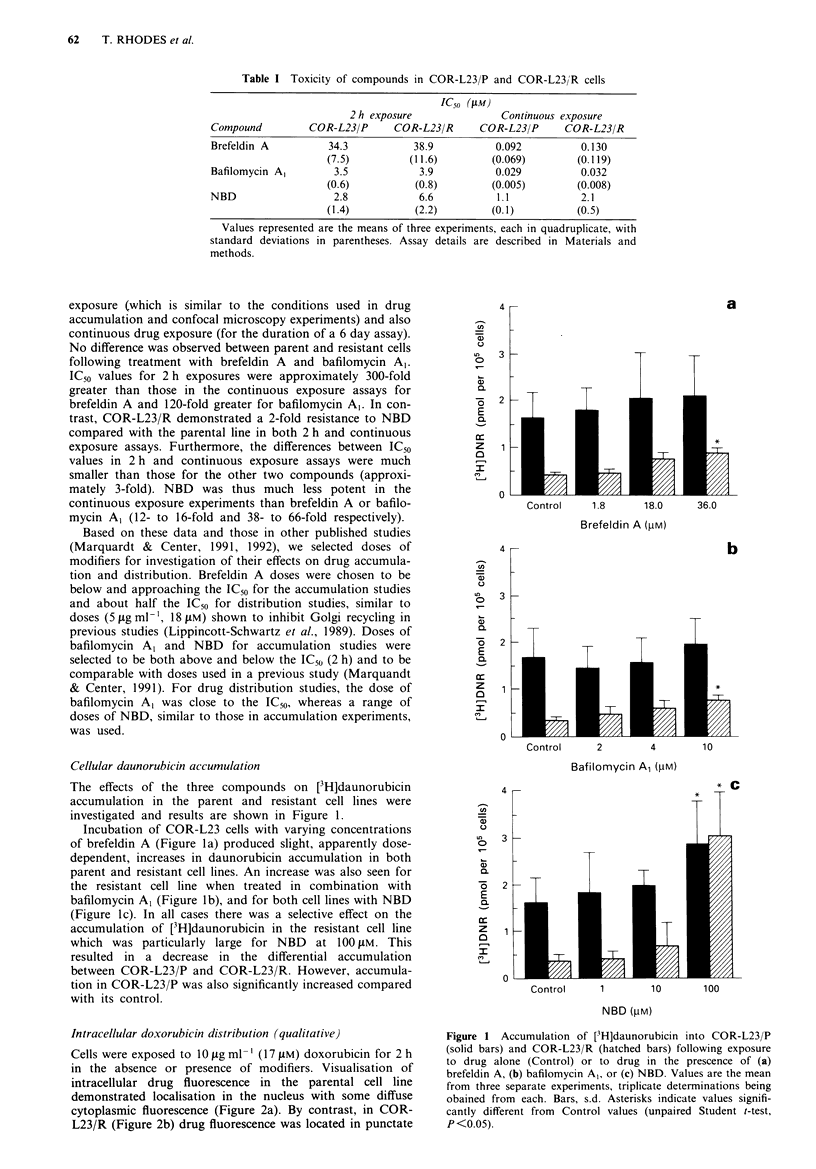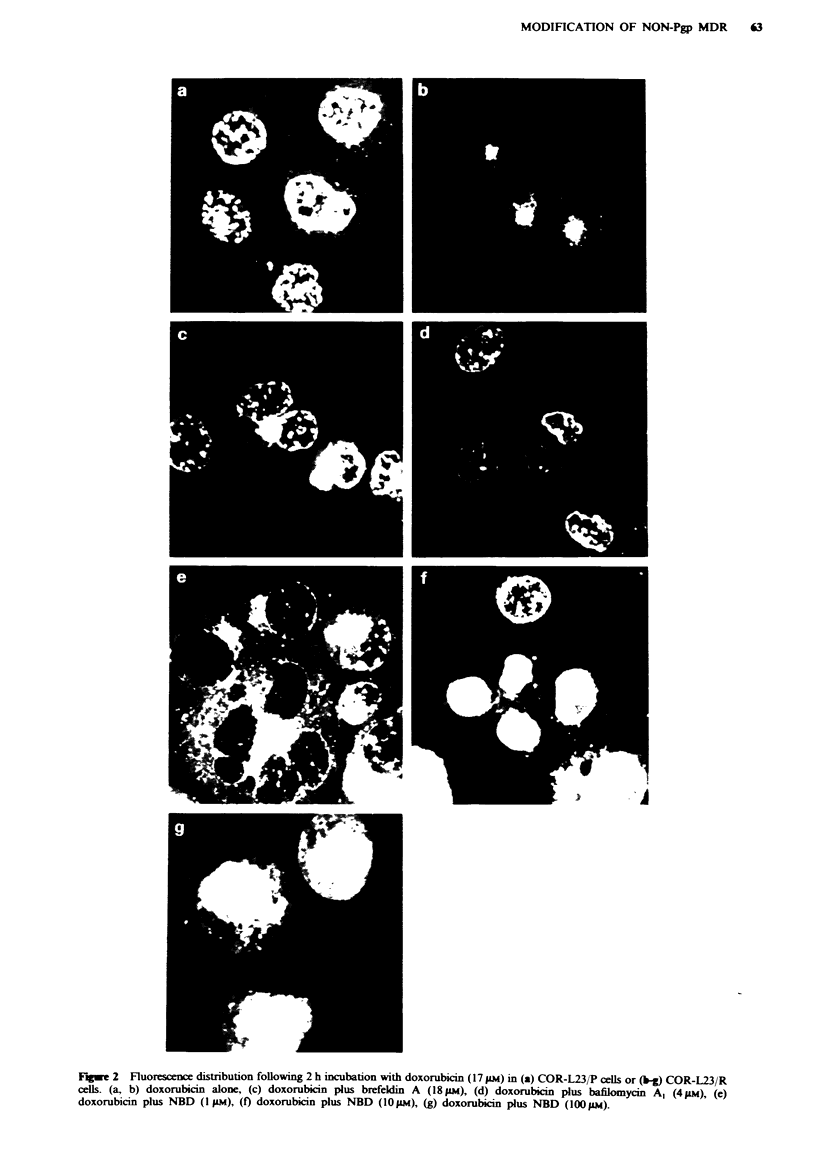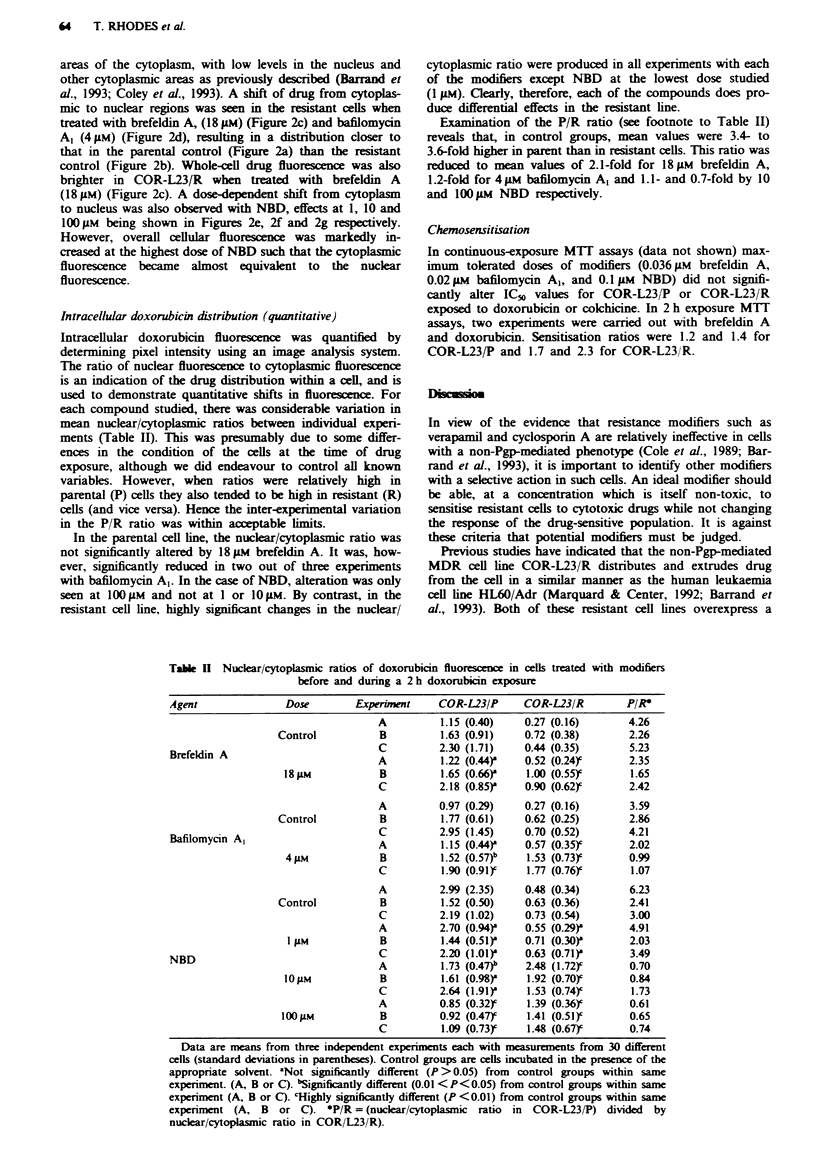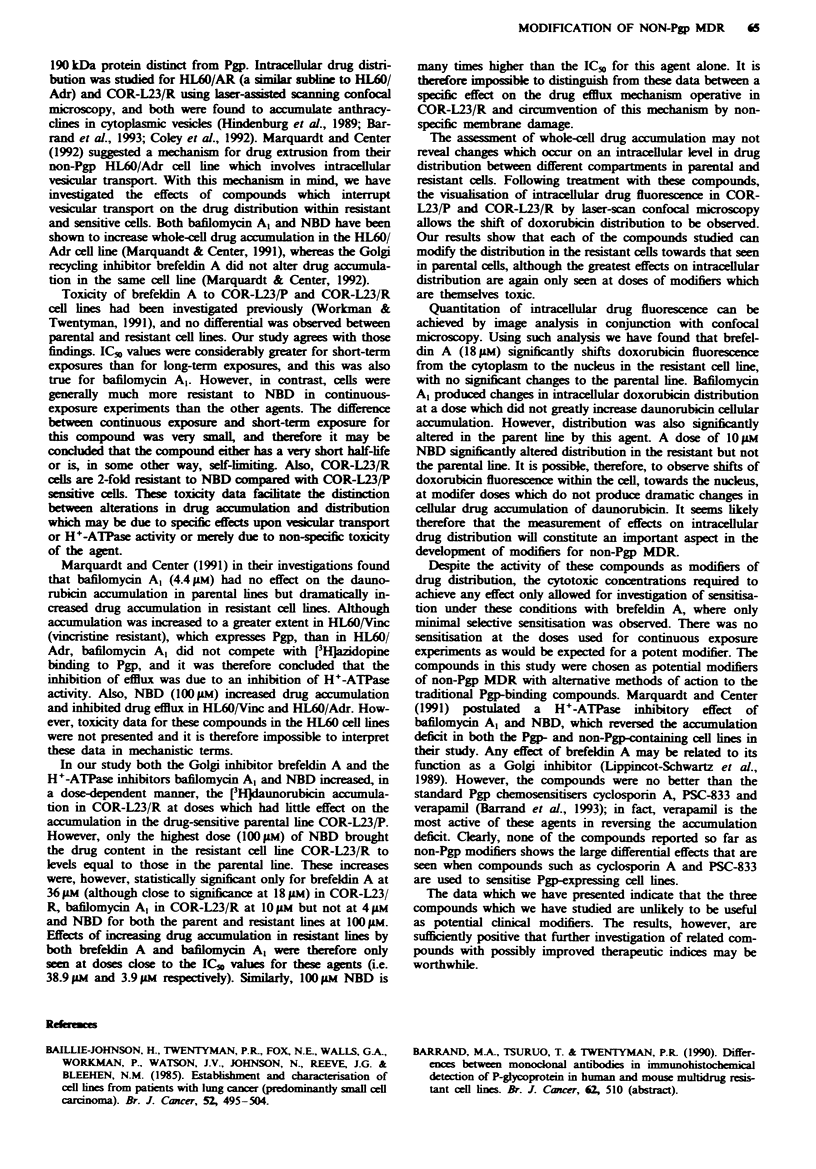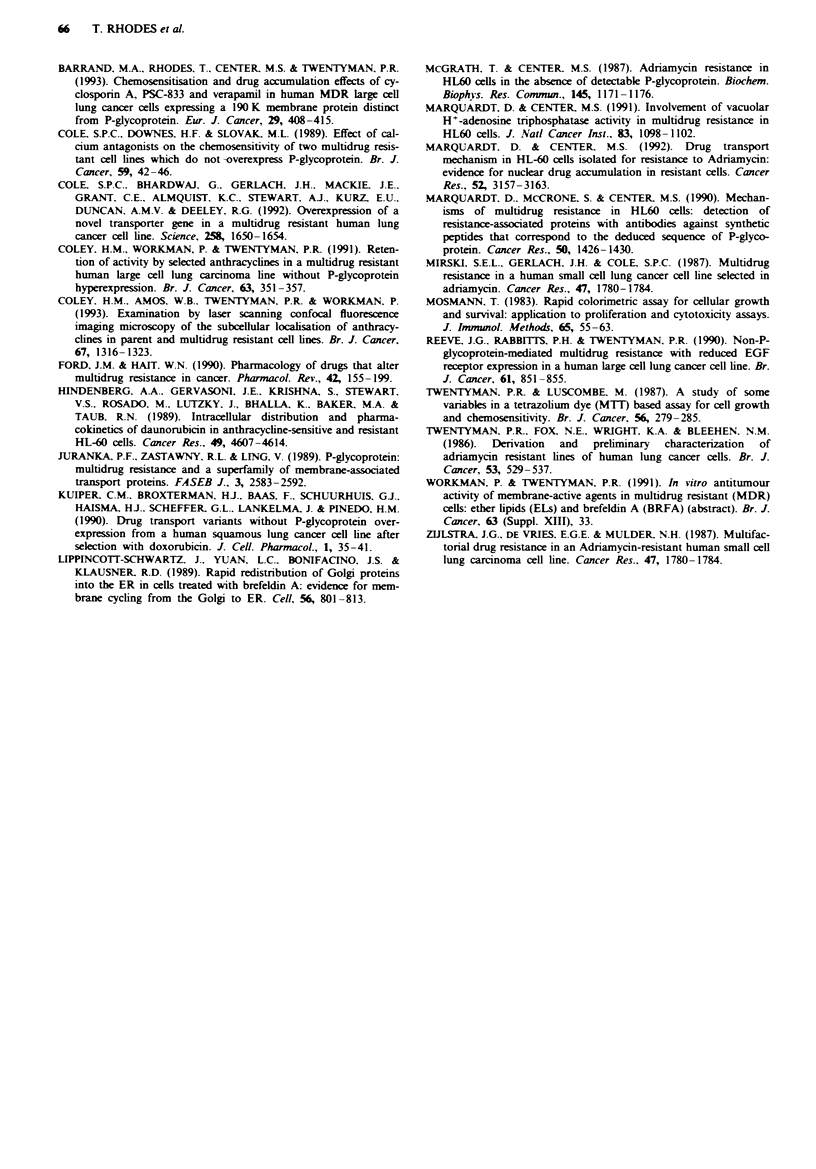# Modification by brefeldin A, bafilomycin A1 and 7-chloro-4-nitrobenz-2-oxa-1,3-diazole (NBD) of cellular accumulation and intracellular distribution of anthracyclines in the non-P-glycoprotein-mediated multidrug-resistant cell line COR-L23/R.

**DOI:** 10.1038/bjc.1994.250

**Published:** 1994-07

**Authors:** T. Rhodes, M. A. Barrand, P. R. Twentyman

**Affiliations:** MRC Clinical Oncology and Radiotherapeutics Unit, MRC Centre, Cambridge, UK.

## Abstract

**Images:**


Br. J. Cancer (1994), 70, 60-66 © Macmillan Press Ltd., 1994

### Modification by brefeldin A, bafilomycin $A_1$ and 7-chloro-4-nitrobenz-2-oxa-1,3-diazole (NBD) of cellular accumulation and intracellular distribution of anthracyclines in the non-P-glycoprotein-mediated multidrug-resistant cell line COR-L23/R

T. Rhodes, M.A. Barrand & P.R. Twentyman

*MRC Clinical Oncology and Radiotherapeutics Unit, MRC Centre, Hills Rd, Cambridge CB2 2QH, UK.*

**Summary** We have investigated the effects of $H^+$-ATPase inhibitors, bafilomycin $A_1$ and 7-chloro-4-nitrobenz-2-oxa-1,3 diazole (NBD), and the Golgi inhibitor, brefeldin A, on daunorubicin accumulation and doxorubicin intracellular distribution in the non-P-glycoprotein-mediated multidrug-resistant cell line COR-L23/R. This cell line overexpresses a 190 kDa protein which is probably the product of the MRP gene and shows an anthracycline accumulation defect and a drastically altered intracellular anthracycline distribution from the parental cell line COR-L23/P. We found that all three agents could selectively increase the cellular accumulation of daunorubicin in resistant cells. However, these effects were only seen at doses of the modifiers which were equal to or greater than the $IC_{50}$ of the modifier alone. Effects of the modifiers on the intracellular distribution of doxorubicin fluorescence could, however, be seen at doses lower than those required to produce significant effects on daunorubicin accumulation. However, when used in a continuous MTT chemosensitivity assay none of the agents, used at maximum non-toxic doses, was able to sensitise COR-L23/R cells to doxorubicin or to colchicine. Although these lead compounds are unlikely to be useful as clinical modifiers, development of more selective analogues may prove useful in the modification of non-P-glycoprotein-mediated multidrug resistance.

Acquired resistance of tumours to a group of structurally and functionally unrelated cytotoxic drugs (multidrug resistance, MDR) can be modelled *in vitro* by exposing tumour cell lines to one of the group of drugs. Overexpression of a 170 kDa glycoprotein (P-glycoprotein, Pgp), acting as an efflux pump in the plasma membrane, has been demonstrated as a principal mechanism of MDR in many such cell lines (Juranka *et al.*, 1989). However, some MDR cell lines efflux MDR drugs but do not overexpress the Pgp pump. An example is the human large-cell lung carcinoma line COR-L23/R, developed by stepwise increases in exposure to doxorubicin (Twentyman *et al.*, 1986), and resistant to doxorubicin, daunorubicin, colchicine, vincristine and VP-16 (Coley *et al.*, 1991). This cell line exhibits decreased intracellular drug accumulation compared with the parental line, with no detectable overexpression of Pgp or of mRNA from the encoding *mdr*1 gene (Barrand *et al.*, 1990; Reeve *et al.*, 1990). A number of other such cell lines with similar patterns of cross-resistance but lacking Pgp have also been described (McGrath & Center, 1987; Mirski *et al.*, 1987; Zijlstra *et al.*, 1987; Kuiper *et al.*, 1990).

A series of antisera to synthetic peptides from the deduced sequence of Pgp were used to study a non-Pgp MDR human leukaemia cell line (HL60/Adr) (Marquardt *et al.*, 1990). One of the antisera detected the presence of a 190 kDa membrane protein in the resistant cells but not in the parental cells from which they were derived. This antiserum (ASP-14) was also used to identify a protein of the same size in our COR-L23/R non-Pgp MDR line (Barrand *et al.*, 1993). It now appears probable that the 190 kDa protein band detected by antiserum ASP14 (and also by our own similar antiserum, CRA-1) includes the product of the MRP gene, isolated and sequenced from a non-Pgp-mediated human small-cell lung cancer MDR subline H69/AR (Cole *et al.*, 1992). The gene sequence indicates that its product bears a close homology to Pgp and is thus likely to play a role as an alternative transporter in cells in which it is overexpressed. It has been shown that compounds such as verapamil and cyclosporin A are highly effective modifiers of drug resistance in MDR cells having overexpression of Pgp (Ford & Hait, 1990). The same compounds, however, have relatively little effect in cells with a non-Pgp-mediated MDR phenotype (Cole *et al.*, 1989; Barrand *et al.*, 1993). It is therefore important to identify other types of compounds which may be more effective as resistance modifiers in cells showing non-Pgp-mediated MDR.

Striking differences in intracellular distribution of anthracyclines have been observed in HL60/Adr and COR-L23/R cells compared with their respective parental cells (Marquardt & Center, 1992; Barrand *et al.*, 1993; Coley *et al.*, 1993). Whereas intracellular fluorescence of drug is mainly confined to the nucleus of COR-L23/P parental cells, the most intensive fluorescence in COR-L23/R resistant cells is seen in groups of perinuclear vesicles. Daunorubicin initially enters the nucleus of HL60/Adr cells, and redistribution follows with efflux of drug from the nucleus of resistant cells. Marquardt *et al.* (1990) have localised the 190 kDa protein in HL60/Adr to the endoplasmic reticulum. They have proposed a pathway of drug extrusion which involves the concentration of anthracyclines into cytoplasmic vesicles followed by an exocytotic process transporting the drug to the exterior of the cell. This proposal is corroborated by evidence that vacuolar $H^+$-ATPase plays an important part in the pathway of drug efflux from these cells (Marquardt & Center, 1991).

We have, therefore, investigated the effect on drug accumulation in our COR-L23/R cell line of the inhibitors of vacuolar $H^+$-ATPases, bafilomycin $A_1$ and 7-chloro-4-nitrobenz-2-oxa-1,3-diazole (NBD), which were reported to restore drug accumulation in the HL60/Adr cells used in the above-mentioned study (Marquardt & Center, 1991). We have also examined the effects of the fungal antibiotic, brefeldin A, which has been identified as an inhibitor of protein recycling from the endoplasmic reticulum into the Golgi body (Lippincott-Schwartz *et al.*, 1989; Marquardt & Center, 1992). The effects of these compounds on the growth, intracellular anthracycline distribution and accumulation and chemosensitisation in COR-L23/P and COR-L23/R cell lines are the focus of this study.

Correspondence: P.R. Twentyman.
Received 23 December 1993; and in revised form 7 March 1994.

### Materials and methods

### *Cell lines*

The large cell lung carcinoma cell line COR-L23/P (Baillie-Johnson *et al.*, 1985) and MDR subline COR-L23/R were derived in this laboratory. COR-L23/R was made drug resistant by *in vitro* exposure to increasing doses of doxorubicin (Twentyman *et al.*, 1986) and is routinely maintained in 0.2 $\mu g\ ml^{-1}$ (0.34 $\mu M$) doxorubicin.

### *Culture conditions*

The cell lines were grown as monolayers in RPMI-1640 medium plus 10% fetal calf serum (both from Gibco Biocult, Paisley, UK). The medium was supplemented with penicillin and streptomycin (100 U $ml^{-1}$ and 100 $\mu g\ ml^{-1}$ respectively) (Gibco Biocult, Paisley, UK). Cell stocks were maintained in 75 $cm^2$ tissue culture flasks (Falcon Plastics, Plymouth, UK) in a humidified atmosphere of 8% carbon dioxide + 92% air at 37°C and were subcultured weekly.

### *Drugs*

Stocks of doxorubicin (Farmitalia Carlo Erba, Milan, Italy) (500 $\mu g\ ml^{-1}$ in water) were kept at −20°C and dilutions were made in PBS before addition to cells. Colchicine (Sigma, Poole, UK) was dissolved in water and kept at −20°C. Solutions of brefeldin A [4-dihydroxy-2-(6-hydroxy-1-heptenyl)-4-cyclopentanecrotonic acid lactone] and NBD (7-chloro-4-nitrobenz-2-oxa-1,3-diazole) (Sigma, Poole, UK) were freshly prepared in ethanol and diluted in RPMI-1640 growth medium before addition to cells. Bafilomycin $A_1$ (Professor K. Altendorf, Universitat Osnabruck, Germany) was dissolved in dimethylsulphoxide (DMSO) and diluted in growth medium before addition to cells. [$^3$H]daunorubicin (specific activity 1.4 Ci $mmol^{-1}$) was obtained from Amersham International.

### *Drug response*

The MTT assay used to determine drug sensitivity was based on that described by Mosmann (1983) and modified in this laboratory (Twentyman & Luscombe, 1987). COR-L23/P and COR-L23/R cells were plated (200 $\mu l$ per well) into 96-well microtitre plates (Falcon Plastics) at $5 \times 10^3$ and $10^4$ cells $ml^{-1}$ respectively. For continuous drug exposure experiments, plates were incubated (8% carbon dioxide, 92% air, 37°C) for 2 h and drugs or appropriate solvent controls added in a volume of 20 $\mu l$. Plates were returned to the incubator for a further 6 days, at which time the assay was carried out. Plates for the 2 h drug exposure were incubated for 24 h after plating. Drugs were then added as previously and removed after 2 h followed by three rinses in warm medium. Fresh medium was then added and the plates returned to the incubator for a further 5 days before the assay was performed. Appropriate solvent controls were used in all experiments. After a total incubation of 6 days, 20 $\mu l$ of MTT (Sigma) (5 mg $ml^{-1}$ in PBS) was added to each well and plates were returned to the incubator for 5 h. Medium was then aspirated, 200 $\mu l$ of DMSO (BDH, Poole, UK) added to each well, and the plates were agitated for 10 min on a plate shaker. Optical densities were read at 540 nm and at a reference wavelength of 690 nm on a Titertek Multiskan MCC ELISA plate reader (Flow Laboratories, Rickmansworth, UK). Results are expressed as a fraction of control. Quadruplicate wells were used at each dose point. $IC_{50}$ values (concentration of agent to reduce final optical density to 50% of control) were read by eye from dose–response curves.

The effects of brefeldin A (0.036 $\mu M$), bafilomycin $A_1$ (0.02 $\mu M$) and NBD (0.1 $\mu M$) on the response of COR-L23/P and COR-L23/R to doxorubicin and colchicine were measured using a continuous 6 day MTT assay as above. Cells were plated onto 96-well plates (as above) and incubated for 2 h, followed by the addition of brefeldin A, bafilomycin $A_1$, or NBD, and, 1 h later, the addition of the cytotoxics doxorubicin and colchicine. Plates were then incubated and response assayed as described in the previous section. The effect of brefeldin A (18 $\mu M$) on the response of COR-L23/P and COR-L23/R to doxorubicin for 2 h was measured by plating cells out and 24 h later adding the brefeldin A, followed 15 min later by the cytotoxic and incubating for 2 h. Plates were then rinsed three times and resuspended in warm medium, incubated for a further 5 days and assayed as above.

### *Daunorubicin accumulation*

COR-L23/P and COR-L23/R cells ($4 \times 10^4$ and $6 \times 10^4$ respectively) were plated in a volume of 2 ml of medium into wells on six-well plates and incubated for 3 days so that the cells were in the logarithmic phase of growth. Growth medium was then aspirated and replaced with fresh medium containing brefeldin A, bafilomycin $A_1$, NBD or solvent control. The cells were then incubated for 15 min before the addition of 0.02 $\mu g\ ml^{-1}$ (0.04 $\mu M$) tritium-labelled daunorubicin and a further incubation for 2 h. Medium was then removed and cells were rinsed three times with ice-cold PBS and lysed with 1 ml of 0.1% SDS. A 0.5 ml aliquot of this solution was transferred into scintillation vials and mixed with 5 ml of Quicksafe A (Zinsser Analytic, Maidenhead, UK). Radioactivity was measured over a period of 5 min on a Beckman LS5000CE. Cells from replicate wells were trypsinised and counted using a haemocytometer in order that results for accumulation could be expressed as drug content per cell.

### *Intracellular doxorubicin distribution*

Cells were plated onto sterile glass coverslips in 2 ml aliquots in Falcon six-well plates at $5 \times 10^4$ $ml^{-1}$ and incubated overnight (8% carbon dioxide, 92% air, at 37°C). The growth medium was then removed and replaced with warm medium containing bafilomycin $A_1$, brefeldin A, NBD or the appropriate solvent. The coverslips were incubated for 15 min, followed by the addition of doxorubicin at 10 $\mu g\ ml^{-1}$ (17 $\mu M$) and further incubation for 2 h. Coverslips were rinsed in ice-cold PBS, inverted onto slides and the edges sealed with Glyceel (Gurr, BDH, Poole, UK) to protect against dehydration. Intracellular doxorubicin fluorescence was observed using the Biorad MRC-600 laserscan confocal microscope (Biorad Lasersharp, Hemel Hempstead, UK) using the 488 nm laser line for excitation in the Biorad BHS filters block, which allows detection of emitted light at all wavelengths above 515 nm. Images were collected and stored on optical discs (Panasonic 470), allowing the measurement of pixel intensities within defined areas of the cells. Ratios of fluorescence within the nucleus and within the cytoplasm were calculated and used to indicate shifts of intracellular drug distribution. Approximately 30 cells were measured for each treatment point in an experiment.

Whereas drug accumulation studies were carried out with daunorubicin because of the ready availability of radiolabelled compound at a reasonable price, we used doxorubicin for confocal studies of intracellular distribution. The reason for this choice is that measurement of nuclear/cytoplasmic fluorescent ratios is facilitated by a better-defined nucleus for doxorubicin than for daunorubicin and because differences between parent and resistant cells are greater for the former anthracycline.

### Results

### *Toxicity of modifiers alone*

MTT assays were carried out in order to assess the toxicity of brefeldin A, bafilomycin $A_1$ and NBD in the COR-L23/P and COR-L23/R cell lines. Effects following different exposure conditions were investigated, and the $IC_{50}$ values are shown in Table I. We used both short-term (i.e. 2 h)

**Table I** Toxicity of compounds in COR-L23/P and COR-L23/R cells

| | $IC_{50}$ (μM) | | | |
|---|---|---|---|---|
| | *2 h exposure* | | *Continuous exposure* | |
| *Compound* | *COR-L23/P* | *COR-L23/R* | *COR-L23/P* | *COR-L23/R* |
| Brefeldin A | 34.3 | 38.9 | 0.092 | 0.130 |
| | (7.5) | (11.6) | (0.069) | (0.119) |
| Bafilomycin $A_1$ | 3.5 | 3.9 | 0.029 | 0.032 |
| | (0.6) | (0.8) | (0.005) | (0.008) |
| NBD | 2.8 | 6.6 | 1.1 | 2.1 |
| | (1.4) | (2.2) | (0.1) | (0.5) |

Values represented are the means of three experiments, each in quadruplicate, with standard deviations in parentheses. Assay details are described in Materials and methods.

exposure (which is similar to the conditions used in drug accumulation and confocal microscopy experiments) and also continuous drug exposure (for the duration of a 6 day assay). No difference was observed between parent and resistant cells following treatment with brefeldin A and bafilomycin $A_1$. $IC_{50}$ values for 2 h exposures were approximately 300-fold greater than those in the continuous exposure assays for brefeldin A and 120-fold greater for bafilomycin $A_1$. In contrast, COR-L23/R demonstrated a 2-fold resistance to NBD compared with the parental line in both 2 h and continuous exposure assays. Furthermore, the differences between $IC_{50}$ values in 2 h and continuous exposure assays were much smaller than those for the other two compounds (approximately 3-fold). NBD was thus much less potent in the continuous exposure experiments than brefeldin A or bafilomycin $A_1$ (12- to 16-fold and 38- to 66-fold respectively).

Based on these data and those in other published studies (Marquardt & Center, 1991, 1992), we selected doses of modifiers for investigation of their effects on drug accumulation and distribution. Brefeldin A doses were chosen to be below and approaching the $IC_{50}$ for the accumulation studies and about half the $IC_{50}$ for distribution studies, similar to doses (5 μg $ml^{-1}$, 18 μM) shown to inhibit Golgi recycling in previous studies (Lippincott-Schwartz *et al.*, 1989). Doses of bafilomycin $A_1$ and NBD for accumulation studies were selected to be both above and below the $IC_{50}$ (2 h) and to be comparable with doses used in a previous study (Marquandt & Center, 1991). For drug distribution studies, the dose of bafilomycin $A_1$ was close to the $IC_{50}$, whereas a range of doses of NBD, similar to those in accumulation experiments, was used.

### *Cellular daunorubicin accumulation*

The effects of the three compounds on [$^3$H]daunorubicin accumulation in the parent and resistant cell lines were investigated and results are shown in Figure 1.

Incubation of COR-L23 cells with varying concentrations of brefeldin A (Figure 1a) produced slight, apparently dose-dependent, increases in daunorubicin accumulation in both parent and resistant cell lines. An increase was also seen for the resistant cell line when treated in combination with bafilomycin $A_1$ (Figure 1b), and for both cell lines with NBD (Figure 1c). In all cases there was a selective effect on the accumulation of [$^3$H]daunorubicin in the resistant cell line which was particularly large for NBD at 100 μM. This resulted in a decrease in the differential accumulation between COR-L23/P and COR-L23/R. However, accumulation in COR-L23/P was also significantly increased compared with its control.

### *Intracellular doxorubicin distribution (qualitative)*

Cells were exposed to 10 μg $ml^{-1}$ (17 μM) doxorubicin for 2 h in the absence or presence of modifiers. Visualisation of intracellular drug fluorescence in the parental cell line demonstrated localisation in the nucleus with some diffuse cytoplasmic fluorescence (Figure 2a). By contrast, in COR-L23/R (Figure 2b) drug fluorescence was located in punctate

**Figure 1** Accumulation of [$^3$H]daunorubicin into COR-L23/P (solid bars) and COR-L23/R (hatched bars) following exposure to drug alone (Control) or to drug in the prescence of (**a**) brefeldin A, (**b**) bafilomycin $A_1$, or (**c**) NBD. Values are the mean from three separate experiments, triplicate determinations being obained from each. Bars, s.d. Asterisks indicate values significantly different from Control values (unpaired Student *t*-test, $P<0.05$).

**Figure 2** Fluorescence distribution following 2 h incubation with doxorubicin (17 μM) in (**a**) COR-L23/P cells or (**b**-**g**) COR-L23/R cells. (a, b) doxorubicin alone, (c) doxorubicin plus brefeldin A (18 μM), (d) doxorubicin plus bafilomycin $A_1$ (4 μM), (e) doxorubicin plus NBD (1 μM), (f) doxorubicin plus NBD (10 μM), (g) doxorubicin plus NBD (100 μM).

areas of the cytoplasm, with low levels in the nucleus and other cytoplasmic areas as previously described (Barrand *et al.*, 1993; Coley *et al.*, 1993). A shift of drug from cytoplasmic to nuclear regions was seen in the resistant cells when treated with brefeldin A, (18 μM) (Figure 2c) and bafilomycin $A_1$ (4 μM) (Figure 2d), resulting in a distribution closer to that in the parental control (Figure 2a) than the resistant control (Figure 2b). Whole-cell drug fluorescence was also brighter in COR-L23/R when treated with brefeldin A (18 μM) (Figure 2c). A dose-dependent shift from cytoplasm to nucleus was also observed with NBD, effects at 1, 10 and 100 μM being shown in Figures 2e, 2f and 2g respectively. However, overall cellular fluorescence was markedly increased at the highest dose of NBD such that the cytoplasmic fluorescence became almost equivalent to the nuclear fluorescence.

### *Intracellular doxorubicin distribution (quantitative)*

Intracellular doxorubicin fluorescence was quantified by determining pixel intensity using an image analysis system. The ratio of nuclear fluorescence to cytoplasmic fluorescence is an indication of the drug distribution within a cell, and is used to demonstrate quantitative shifts in fluorescence. For each compound studied, there was considerable variation in mean nuclear/cytoplasmic ratios between individual experiments (Table II). This was presumably due to some differences in the condition of the cells at the time of drug exposure, although we did endeavour to control all known variables. However, when ratios were relatively high in parental (P) cells they also tended to be high in resistant (R) cells (and vice versa). Hence the inter-experimental variation in the P/R ratio was within acceptable limits.

In the parental cell line, the nuclear/cytoplasmic ratio was not significantly altered by 18 μM brefeldin A. It was, however, significantly reduced in two out of three experiments with bafilomycin $A_1$. In the case of NBD, alteration was only seen at 100 μM and not at 1 or 10 μM. By contrast, in the resistant cell line, highly significant changes in the nuclear/cytoplasmic ratio were produced in all experiments with each of the modifiers except NBD at the lowest dose studied (1 μM). Clearly, therefore, each of the compounds does produce differential effects in the resistant line.

Examination of the P/R ratio (see footnote to Table II) reveals that, in control groups, mean values were 3.4- to 3.6-fold higher in parent than in resistant cells. This ratio was reduced to mean values of 2.1-fold for 18 μM brefeldin A, 1.2-fold for 4 μM bafilomycin $A_1$ and 1.1- and 0.7-fold by 10 and 100 μM NBD respectively.

### *Chemosensitisation*

In continuous-exposure MTT assays (data not shown) maximum tolerated doses of modifiers (0.036 μM brefeldin A, 0.02 μM bafilomycin $A_1$, and 0.1 μM NBD) did not significantly alter $IC_{50}$ values for COR-L23/P or COR-L23/R exposed to doxorubicin or colchicine. In 2 h exposure MTT assays, two experiments were carried out with brefeldin A and doxorubicin. Sensitisation ratios were 1.2 and 1.4 for COR-L23/P and 1.7 and 2.3 for COR-L23/R.

### Discussion

In view of the evidence that resistance modifiers such as verapamil and cyclosporin A are relatively ineffective in cells with a non-Pgp-mediated phenotype (Cole *et al.*, 1989; Barrand *et al.*, 1993), it is important to identify other modifiers with a selective action in such cells. An ideal modifier should be able, at a concentration which is itself non-toxic, to sensitise resistant cells to cytotoxic drugs while not changing the response of the drug-sensitive population. It is against these criteria that potential modifiers must be judged.

Previous studies have indicated that the non-Pgp-mediated MDR cell line COR-L23/R distributes and extrudes drug from the cell in a similar manner as the human leukaemia cell line HL60/Adr (Marquard & Center, 1992; Barrand *et al.*, 1993). Both of these resistant cell lines overexpress a

**Table II** Nuclear/cytoplasmic ratios of doxorubicin fluorescence in cells treated with modifiers before and during a 2 h doxorubicin exposure

| *Agent* | *Dose* | *Experiment* | *COR-L23/P* | *COR-L23/R* | *P/R** |
|---|---|---|---|---|---|
| Brefeldin A | Control | A | 1.15 (0.40) | 0.27 (0.16) | 4.26 |
| | | B | 1.63 (0.91) | 0.72 (0.38) | 2.26 |
| | | C | 2.30 (1.71) | 0.44 (0.35) | 5.23 |
| | 18 μM | A | 1.22 (0.44)[a] | 0.52 (0.24)[c] | 2.35 |
| | | B | 1.65 (0.66)[a] | 1.00 (0.55)[c] | 1.65 |
| | | C | 2.18 (0.85)[a] | 0.90 (0.62)[c] | 2.42 |
| Bafilomycin $A_1$ | Control | A | 0.97 (0.29) | 0.27 (0.16) | 3.59 |
| | | B | 1.77 (0.61) | 0.62 (0.25) | 2.86 |
| | | C | 2.95 (1.45) | 0.70 (0.52) | 4.21 |
| | 4 μM | A | 1.15 (0.44)[a] | 0.57 (0.35)[c] | 2.02 |
| | | B | 1.52 (0.57)[b] | 1.53 (0.73)[c] | 0.99 |
| | | C | 1.90 (0.91)[c] | 1.77 (0.76)[c] | 1.07 |
| NBD | Control | A | 2.99 (2.35) | 0.48 (0.34) | 6.23 |
| | | B | 1.52 (0.50) | 0.63 (0.36) | 2.41 |
| | | C | 2.19 (1.02) | 0.73 (0.54) | 3.00 |
| | 1 μM | A | 2.70 (0.94)[a] | 0.55 (0.29)[a] | 4.91 |
| | | B | 1.44 (0.51)[a] | 0.71 (0.30)[a] | 2.03 |
| | | C | 2.20 (1.01)[a] | 0.63 (0.71)[a] | 3.49 |
| | 10 μM | A | 1.73 (0.47)[b] | 2.48 (1.72)[c] | 0.70 |
| | | B | 1.61 (0.98)[a] | 1.92 (0.70)[c] | 0.84 |
| | | C | 2.64 (1.91)[a] | 1.53 (0.74)[c] | 1.73 |
| | 100 μM | A | 0.85 (0.32)[c] | 1.39 (0.36)[c] | 0.61 |
| | | B | 0.92 (0.47)[c] | 1.41 (0.51)[c] | 0.65 |
| | | C | 1.09 (0.73)[c] | 1.48 (0.67)[c] | 0.74 |

Data are means from three independent experiments each with measurements from 30 different cells (standard deviations in parentheses). Control groups are cells incubated in the presence of the appropriate solvent. [a]Not significantly different ($P > 0.05$) from control groups within same experiment. (A, B or C). [b]Significantly different ($0.01 < P < 0.05$) from control groups within same experiment (A, B or C). [c]Highly significantly different ($P < 0.01$) from control groups within same experiment (A, B or C). *P/R = (nuclear/cytoplasmic ratio in COR-L23/P) divided by nuclear/cytoplasmic ratio in COR/L23/R).

190 kDa protein distinct from Pgp. Intracellular drug distribution was studied for HL60/AR (a similar subline to HL60/Adr) and COR-L23/R using laser-assisted scanning confocal microscopy, and both were found to accumulate anthracyclines in cytoplasmic vesicles (Hindenburg *et al.*, 1989; Barrand *et al.*, 1993; Coley *et al.*, 1992). Marquardt and Center (1992) suggested a mechanism for drug extrusion from their non-Pgp HL60/Adr cell line which involves intracellular vesicular transport. With this mechanism in mind, we have investigated the effects of compounds which interrupt vesicular transport on the drug distribution within resistant and sensitive cells. Both bafilomycin $A_1$ and NBD have been shown to increase whole-cell drug accumulation in the HL60/Adr cell line (Marquandt & Center, 1991), whereas the Golgi recycling inhibitor brefeldin A did not alter drug accumulation in the same cell line (Marquardt & Center, 1992).

Toxicity of brefeldin A to COR-L23/P and COR-L23/R cell lines had been investigated previously (Workman & Twentyman, 1991), and no differential was observed between parental and resistant cell lines. Our study agrees with those findings. $IC_{50}$ values were considerably greater for short-term exposures than for long-term exposures, and this was also true for bafilomycin $A_1$. However, in contrast, cells were generally much more resistant to NBD in continuous-exposure experiments than the other agents. The difference between continuous exposure and short-term exposure for this compound was very small, and therefore it may be concluded that the compound either has a very short half-life or is, in some other way, self-limiting. Also, COR-L23/R cells are 2-fold resistant to NBD compared with COR-L23/P sensitive cells. These toxicity data facilitate the distinction between alterations in drug accumulation and distribution which may be due to specific effects upon vesicular transport or $H^+$-ATPase activity or merely due to non-specific toxicity of the agent.

Marquardt and Center (1991) in their investigations found that bafilomycin $A_1$ (4.4 μM) had no effect on the daunorubicin accumulation in parental lines but dramatically increased drug accumulation in resistant cell lines. Although accumulation was increased to a greater extent in HL60/Vinc (vincristine resistant), which expresses Pgp, than in HL60/Adr, bafilomycin $A_1$ did not compete with [$^3$H]azidopine binding to Pgp, and it was therefore concluded that the inhibition of efflux was due to an inhibition of $H^+$-ATPase activity. Also, NBD (100 μM) increased drug accumulation and inhibited drug efflux in HL60/Vinc and HL60/Adr. However, toxicity data for these compounds in the HL60 cell lines were not presented and it is therefore impossible to interpret these data in mechanistic terms.

In our study both the Golgi inhibitor brefeldin A and the $H^+$-ATPase inhibitors bafilomycin $A_1$ and NBD increased, in a dose-dependent manner, the [$^3$H]daunorubicin accumulation in COR-L23/R at doses which had little effect on the accumulation in the drug-sensitive parental line COR-L23/P. However, only the highest dose (100 μM) of NBD brought the drug content in the resistant cell line COR-L23/R to levels equal to those in the parental line. These increases were, however, statistically significant only for brefeldin A at 36 μM (although close to significance at 18 μM) in COR-L23/R, bafilomycin $A_1$ in COR-L23/R at 10 μM but not at 4 μM and NBD for both the parent and resistant lines at 100 μM. Effects of increasing drug accumulation in resistant lines by both brefeldin A and bafilomycin $A_1$ were therefore only seen at doses close to the $IC_{50}$ values for these agents (i.e. 38.9 μM and 3.9 μM respectively). Similarly, 100 μM NBD is many times higher than the $IC_{50}$ for this agent alone. It is therefore impossible to distinguish from these data between a specific effect on the drug efflux mechanism operative in COR-L23/R and circumvention of this mechanism by non-specific membrane damage.

The assessment of whole-cell drug accumulation may not reveal changes which occur on an intracellular level in drug distribution between different compartments in parental and resistant cells. Following treatment with these compounds, the visualisation of intracellular drug fluorescence in COR-L23/P and COR-L23/R by laser-scan confocal microscopy allows the shift of doxorubicin distribution to be observed. Our results show that each of the compounds studied can modify the distribution in the resistant cells towards that seen in parental cells, although the greatest effects on intracellular distribution are again only seen at doses of modifiers which are themselves toxic.

Quantitation of intracellular drug fluorescence can be achieved by image analysis in conjunction with confocal microscopy. Using such analysis we have found that brefeldin A (18 μM) significantly shifts doxorubicin fluorescence from the cytoplasm to the nucleus in the resistant cell line, with no significant changes to the parental line. Bafilomycin $A_1$ produced changes in intracellular doxorubicin distribution at a dose which did not greatly increase daunorubicin cellular accumulation. However, distribution was also significantly altered in the parent line by this agent. A dose of 10 μM NBD significantly altered distribution in the resistant but not the parental line. It is possible, therefore, to observe shifts of doxorubicin fluorescence within the cell, towards the nucleus, at modifer doses which do not produce dramatic changes in cellular drug accumulation of daunorubicin. It seems likely therefore that the measurement of effects on intracellular drug distribution will constitute an important aspect in the development of modifiers for non-Pgp MDR.

Despite the activity of these compounds as modifiers of drug distribution, the cytotoxic concentrations required to achieve any effect only allowed for investigation of sensitisation under these conditions with brefeldin A, where only minimal selective sensitisation was observed. There was no sensitisation at the doses used for continuous exposure experiments as would be expected for a potent modifier. The compounds in this study were chosen as potential modifiers of non-Pgp MDR with alternative methods of action to the traditional Pgp-binding compounds. Marquardt and Center (1991) postulated a $H^+$-ATPase inhibitory effect of bafilomycin $A_1$ and NBD, which reversed the accumulation deficit in both the Pgp- and non-Pgp-containing cell lines in their study. Any effect of brefeldin A may be related to its function as a Golgi inhibitor (Lippincot-Schwartz *et al.*, 1989). However, the compounds were no better than the standard Pgp chemosensitisers cyclosporin A, PSC-833 and verapamil (Barrand *et al.*, 1993); in fact, verapamil is the most active of these agents in reversing the accumulation deficit. Clearly, none of the compounds reported so far as non-Pgp modifiers shows the large differential effects that are seen when compounds such as cyclosporin A and PSC-833 are used to sensitise Pgp-expressing cell lines.

The data which we have presented indicate that the three compounds which we have studied are unlikely to be useful as potential clinical modifiers. The results, however, are sufficiently positive that further investigation of related compounds with possibly improved therapeutic indices may be worthwhile.

### References

BAILLIE-JOHNSON, H., TWENTYMAN, P.R., FOX, N.E., WALLS, G.A., WORKMAN, P., WATSON, J.V., JOHNSON, N., REEVE, J.G. & BLEEHEN, N.M. (1985). Establishment and characterisation of cell lines from patients with lung cancer (predominantly small cell carcinoma). *Br. J. Cancer*, **52**, 495–504.

BARRAND, M.A., TSURUO, T. & TWENTYMAN, P.R. (1990). Differences between monoclonal antibodies in immunohistochemical detection of P-glycoprotein in human and mouse multidrug resistant cell lines. *Br. J. Cancer*, **62**, 510 (abstract).

BARRAND, M.A., RHODES, T., CENTER, M.S. & TWENTYMAN, P.R. (1993). Chemosensitisation and drug accumulation effects of cyclosporin A, PSC-833 and verapamil in human MDR large cell lung cancer cells expressing a 190 K membrane protein distinct from P-glycoprotein. *Eur. J. Cancer*, **29**, 408–415.

COLE, S.P.C., DOWNES, H.F. & SLOVAK, M.L. (1989). Effect of calcium antagonists on the chemosensitivity of two multidrug resistant cell lines which do not overexpress P-glycoprotein. *Br. J. Cancer*, **59**, 42–46.

COLE, S.P.C., BHARDWAJ, G., GERLACH, J.H., MACKIE, J.E., GRANT, C.E., ALMQUIST, K.C., STEWART, A.J., KURZ, E.U., DUNCAN, A.M.V. & DEELEY, R.G. (1992). Overexpression of a novel transporter gene in a multidrug resistant human lung cancer cell line. *Science*, **258**, 1650–1654.

COLEY, H.M., WORKMAN, P. & TWENTYMAN, P.R. (1991). Retention of activity by selected anthracyclines in a multidrug resistant human large cell lung carcinoma line without P-glycoprotein hyperexpression. *Br. J. Cancer*, **63**, 351–357.

COLEY, H.M., AMOS, W.B., TWENTYMAN, P.R. & WORKMAN, P. (1993). Examination by laser scanning confocal fluorescence imaging microscopy of the subcellular localisation of anthracyclines in parent and multidrug resistant cell lines. *Br. J. Cancer*, **67**, 1316–1323.

FORD, J.M. & HAIT, W.N. (1990). Pharmacology of drugs that alter multidrug resistance in cancer. *Pharmacol. Rev.*, **42**, 155–199.

HINDENBERG, A.A., GERVASONI, J.E., KRISHNA, S., STEWART, V.S., ROSADO, M., LUTZKY, J., BHALLA, K., BAKER, M.A. & TAUB, R.N. (1989). Intracellular distribution and pharmacokinetics of daunorubicin in anthracycline-sensitive and resistant HL-60 cells. *Cancer Res.*, **49**, 4607–4614.

JURANKA, P.F., ZASTAWNY, R.L. & LING, V. (1989). P-glycoprotein: multidrug resistance and a superfamily of membrane-associated transport proteins. *FASEB J.*, **3**, 2583–2592.

KUIPER, C.M., BROXTERMAN, H.J., BAAS, F., SCHUURHUIS, G.J., HAISMA, H.J., SCHEFFER, G.L., LANKELMA, J. & PINEDO, H.M. (1990). Drug transport variants without P-glycoprotein overexpression from a human squamous lung cancer cell line after selection with doxorubicin. *J. Cell. Pharmacol.*, **1**, 35–41.

LIPPINCOTT-SCHWARTZ, J., YUAN, L.C., BONIFACINO, J.S. & KLAUSNER, R.D. (1989). Rapid redistribution of Golgi proteins into the ER in cells treated with brefeldin A: evidence for membrane cycling from the Golgi to ER. *Cell*, **56**, 801–813.

McGRATH, T. & CENTER, M.S. (1987). Adriamycin resistance in HL60 cells in the absence of detectable P-glycoprotein. *Biochem. Biophys. Res. Commun.*, **145**, 1171–1176.

MARQUARDT, D. & CENTER, M.S. (1991). Involvement of vacuolar $H^+$-adenosine triphosphatase activity in multidrug resistance in HL60 cells. *J. Natl Cancer Inst.*, **83**, 1098–1102.

MARQUARDT, D. & CENTER, M.S. (1992). Drug transport mechanism in HL-60 cells isolated for resistance to Adriamycin: evidence for nuclear drug accumulation in resistant cells. *Cancer Res.*, **52**, 3157–3163.

MARQUARDT, D., McCRONE, S. & CENTER, M.S. (1990). Mechanisms of multidrug resistance in HL60 cells: detection of resistance-associated proteins with antibodies against synthetic peptides that correspond to the deduced sequence of P-glycoprotein. *Cancer Res.*, **50**, 1426–1430.

MIRSKI, S.E.L., GERLACH, J.H. & COLE, S.P.C. (1987). Multidrug resistance in a human small cell lung cancer cell line selected in adriamycin. *Cancer Res.*, **47**, 1780–1784.

MOSMANN, T. (1983). Rapid colorimetric assay for cellular growth and survival: application to proliferation and cytotoxicity assays. *J. Immunol. Methods*, **65**, 55–63.

REEVE, J.G., RABBITTS, P.H. & TWENTYMAN, P.R. (1990). Non-P-glycoprotein-mediated multidrug resistance with reduced EGF receptor expression in a human large cell lung cancer cell line. *Br. J. Cancer*, **61**, 851–855.

TWENTYMAN, P.R. & LUSCOMBE, M. (1987). A study of some variables in a tetrazolium dye (MTT) based assay for cell growth and chemosensitivity. *Br. J. Cancer*, **56**, 279–285.

TWENTYMAN, P.R., FOX, N.E., WRIGHT, K.A. & BLEEHEN, N.M. (1986). Derivation and preliminary characterization of adriamycin resistant lines of human lung cancer cells. *Br. J. Cancer*, **53**, 529–537.

WORKMAN, P. & TWENTYMAN, P.R. (1991). *In vitro* antitumour activity of membrane-active agents in multidrug resistant (MDR) cells: ether lipids (ELs) and brefeldin A (BRFA) (abstract). *Br. J. Cancer*, **63** (Suppl. XIII), 33.

ZIJLSTRA, J.G., DE VRIES, E.G.E. & MULDER, N.H. (1987). Multifactorial drug resistance in an Adriamycin-resistant human small cell lung carcinoma cell line. *Cancer Res.*, **47**, 1780–1784.